# Challenging PD-L1 expressing cytotoxic T cells as a predictor for response to immunotherapy in melanoma

**DOI:** 10.1038/s41467-018-05047-1

**Published:** 2018-07-26

**Authors:** Lieve Brochez, Annabel Meireson, Inès Chevolet, Nora Sundahl, Piet Ost, Vibeke Kruse

**Affiliations:** 1 Department of Dermatology, Ghent University Hospital, Corneel Heymanslaan 10, 9000 Ghent, Belgium; 2 Dermatology Research Unit, Ghent University Hospital, Corneel Heymanslaan 10, 9000 Ghent, Belgium; 3 Immuno-Oncology Network Ghent (ION Ghent), Ghent, Belgium; 4 Cancer Research Institute Ghent (CRIG), Ghent, Belgium; 5 Department of Radiotherapy, Ghent University Hospital, Corneel Heymanslaan 10, 9000 Ghent, Belgium; 6 Department of Medical Oncology, Ghent University Hospital, Corneel Heymanslaan 10, 9000 Ghent, Belgium

## Introduction

We read with great interest the report by Jacquelot N et al.^1^ on ‘predictors of responses to immune checkpoint blockade in advanced melanoma’. Based on an ex vivo metastatic lymph node assay (stage III, *n* = 37), the authors conclude that PD-L1 expression on circulating CD4^+^ and CD8^+^ T cells might be predictive biomarkers for resistance to CTLA-4 blockade. These observations were confirmed in vivo in another patient cohort, including 190 unresectable stage III and IV melanoma patients who were treated with ipilimumab.

We would like to report our observation that PD-L1 expressing cytotoxic T cells are associated with an altered immune climate and a negative impact on disease outcome in melanoma patients independent of treatment. It therefore meets the criteria of a prognostic biomarker^2^. Whether this biomarker is also predictive for response to immunotherapy—meaning that the effect of immunotherapy is different in biomarker positive and negative patients—cannot be deduced from the retrospective single-arm (only treated patients) data in the study by Jacquelot et al. In fact this would require at least a two-arm design preferably in a randomized study, and known PD-L1^+^ cytotoxic T levels in all patients. The only way to permit solid conclusions on its predictive value would be to demonstrate that the treatment-by-biomarker interaction is statistically significant, meaning that the levels of PD-L1 cytotoxic T cells are not only associated with worse disease outcome (prognostic), but are also associated with different response reactions to immunotherapy (predictive).

We investigated the in vivo immune profile in a cohort of 55 stage I to III patients having undergone only surgery. The median follow-up after diagnosis was 61 months (IQR: 38–112). During follow-up 15 patients (27.3%) experienced disease relapse and six patients (10.9%) died. Blood sampling for immune profiling was performed at a median time of 15 months (IQR: 4–60) after diagnosis. Patients’ characteristics are presented in Supplementary Table 1.

The level of PD-L1^+ ^CD8^+^ cells was strongly correlated with a number of markers indicating a negative immune climate (Table 1). In the current study cohort, a high frequency of PD-L1^+ ^CD8^+^ cells was associated with decreased plasmacytoid dendritic cells (pDCs) and increased myeloid-derived suppressor cells (MDSCs). We previously demonstrated that these cell types are inversely correlated and have an independent prognostic effect on overall survival (OS) in melanoma patients^3^. Although PD-L1^+ ^CD8^+^ cells were negatively correlated with CD4^+^ cells there was a strong correlation with Tregs (as detected by CD3^+^ CD4^+^ CD25^+^ FoxP3^+^ expression) and the number of CTLA-4 expressing Tregs. On the other hand, levels of PD-L1^+ ^CD8^+^ cells were positively correlated with the serum kynurenine/tryptophan (kyn/trp) ratio, reflecting increased functional activity of indoleamine 2, 3-dioxygenase (IDO) in these patients. The intracellular enzyme IDO degrades tryptophan to kynurenine which is considered to be a mechanism attributing to immune tolerance. IDO has been demonstrated to be a negative prognostic marker in a variety of cancers^4^. The above results imply that PD-L1 expression on cytotoxic T-cells is in close interplay with other immune-inhibitory cell types and molecules, all together contributing to the installation of a negative immune climate.

**Table 1:** Association of PD-L1^+ ^CD8^+^ cells with other systemic immune markers

	Spearman correlation coefficient	*P*-value
**Serum**		
Kyn/Trp	0.339	0.014
**PBMCs: T-lymphocytes**		
CD3^+^	0.367	0.006
CD4^+^	−0.446	0.001
Tregs	0.414	0.002
CTLA-4^+ ^Tregs	0.399	0.003
CD8^+^	−0.589	<0.001
**PBMCs: dendritic cells**		
MDSC/pDC	0.304	0.024

**Fig. 1:**
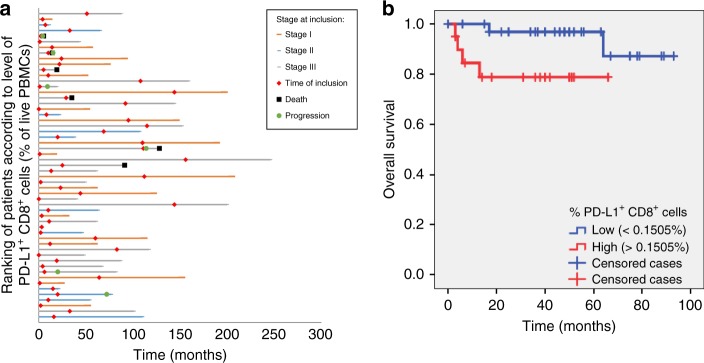
PD-L1^+^ CD8^+^ level according to disease stage and overall survival. Swimmer plot showing the relation between the ranking of PD-L1^+ ^CD8^+^ cells (according to frequency) and clinical follow-up. **b** Kaplan–Meier curves of overall survival according to the levels of PD-L1^+ ^CD8^+^ cells (% of live peripheral blood mononuclear cells)

The clinical follow-up of the included patients is illustrated in Fig. 1a. Patients who died from melanoma (*n* = 6; median time of 9.5 months after blood sampling, IQR: 3.8–28.8) had higher levels of PD-L1^+ ^CD8^+^ cells (*p* = 0.039, Mann–Whitney *U*-test). For survival analysis, the percentage of circulating PD-L1^+ ^CD8^+^ cells was dichotomized by ROC analysis into “low” (<0.1505% of live PBMCs) and “high” (>0.1505% of live PBMCs). High levels of PD-L1^+ ^CD8^+^ cells were observed to be a negative prognostic marker for OS in univariate analysis (*p* = 0.038, log-rank test, Fig. 1b). Multivariate analysis with a Cox proportional hazard regression model including disease stage (local versus regional disease) demonstrated PD-L1^+ ^CD8^+^ cells to be an independent negative prognostic marker on OS (*p* = 0.047; HR: 7.42, 95% CI: 1.03–53.67). Moreover, the frequency of PD-L1^+ ^CD8^+^ cells was increased in patients who were close to disease relapse or disease related death. The shorter the time frame between blood sampling and relapse or death, the higher the frequency of PD-L1^+^  CD8^+^ cells (resp. *p* = 0.005, CC −0.975 and *p* = 0.005, CC −0.943).

Jacquelot et al. state that prediction of patients’ response to immunotherapy by dynamic biomarkers represent an unmet medical need. More important than finding predictors for immunotherapy (no-) response may be the development of tools to routinely monitor the immune response before and during immunotherapy. If negative immunological signs are observed, there might be an opportunity for early intervention to try to reverse this climate. In 10 stage IV melanoma patients treated with ipilimumab and stereotactic body radiotherapy, non-responders showed an increase in the kyn/trp ratio during treatment compared to baseline^5^. Wang et al.^6^ recently demonstrated that increasing kyn/trp ratio after chemoradiation predicts worse survival outcome in lung cancer patients. As the kyn/trp ratio is reflecting IDO activity, these patients subgroups might be candidates for an IDO-inhibitor.

Immunomonitoring before and during cancer (immuno-) therapy, and sequential therapy adapted to the observations—rather than upfront combinations—may be a new step in the era of personalized immunotherapy.

## Methods

### Patients

Fifty-five patients with melanoma were included in this study, with a median follow-up of 61 months (38.0–112.0) after diagnosis and 46 months (22.0–55.0) after inclusion (i.e., the time of blood collection). Only patients who did not receive systemic therapy were included, to avoid any possible influence on marker expression and survival analysis. Detailed patient characteristics can be found in Supplementary Table 1. This study was approved by the ethical committee of Ghent University Hospital. All included patients provided informed consent before enrollment in the study.

### PBMC isolation

Peripheral blood mononuclear cells (PBMCs) were isolated from heparinized venous blood by centrifugation on a Ficoll-Hypaque gradient (GE Healthcare, Uppsala, Sweden) within a 4 h of venipuncture. The cells were washed three times and cryopreserved at −80 °C in fetal bovine serum (FBS, Invitrogen, Merelbeke, Gelbium) supplemented with 10% dimethyl sulfoxide (DMSO, Merck, Darmstadt, Germany) and 1% penicillin-streptavidine (Invitrogen, Merelbeke, Belgium) until analysis. Cells were thawed by submersion at 37° for 1–2 min and resuspended in a medium containing Iscove’s Modified Dulbeco’s Medium (IMDM) supplemented with 20% FBS and 1% glutamine.

### Flow cytometry

MDSCs were characterized by the HLA-DR^–^ lineage^–^ (CD3, CD19, and CD56) CD33^+^ CD11b^+^ phenotype. Dendritic cells were characterized by the HLA-DR^+^ lineage^–^ (CD3, CD14, CD16, CD19, CD20, and CD56) phenotype, pDCs are CD123^+^ CD11c^‒^. Tregs were defined as CD3^+^ CD4^+^ CD25^+^ FoxP3^+^ and cytotoxic T-cells as CD3^+^ CD8^+^ cells. All antibodies used in this study were fluorescently conjugated mouse anti-human monoclonal antibodies. The following antibodies were purchased from BD Biosciences; CD3 BV421 (563797), CD4 APC-Cy7 (561839), CD25 FITC (560990), CD33 BV421 (562854), CD11b APC-Cy7 (560914), CD123 BV421 (562517). The following antibodies were purchased from eBioscience; B7-H1 PE-Cy7 (25-5983-42), CD8 APC (9017-0087-025), CD3 FITC (11-0038-41), CD19 FITC (11-0199-41), CD56 FITC (11-0569-41), CD14 APC (17-0149-41), CD11c APC (17-0116-41), HLA-DR PerCP-Cy5.5 (45-9956-42). For intracellular stainings, after surface staining PBMCs were fixed and permeabilized with fixation⁄permeabilization solution (BD Biosciences), and then stained with CTLA-4 APC (BD Biosciences, 560938) and FoxP3 PerCP-Cy5.5 (eBioscience, 45-4776-42) antibodies. Live/dead staining was performed using Live/dead® fixable aqua dead cell stain (Life Technologies Europe). Cells were analyzed on a FACSCanto™ II flow cytometer (BD Bioscience, Erembodegem, Belgium) using FlowJo software (Tree Star Inc, Ashland, OR, USA). For setting the gates, isotype, and fluorescence-minus-one (FMO) controls were used. To provide a representative sample a median amount of 500,000 cells was analysed per cell type (min 261,000–max 569,750). The reported frequencies of circulating cell types are percentages of live PBMCs, except for Treg frequencies which are percentages of CD4^+^ cells.

### Ultra-performance liquid chromatography (UPLC)

UPLC-MS/MS (Waters Acquity TQD) was conducted to quantify tryptophan and kynurenin on frozen patient sera, according to previously published methods^7^.

### Statistical analysis

All statistical analyses were performed using SPSS 24.0 (SPSS Inc, Chicago, IL, USA), a *P*-value less than 0.05 was considered statistically significant (double-sided). Shapiro-Wilk test demonstrated that the data had no normal distribution and therefore non-parametric statistical tests were used for further analysis. Spearman correlation coefficients (CC) were calculated to evaluate correlations between continuous variables.. Values between two groups were compared by a two-tailed Mann–Whitney *U*-test. OS was estimated by the Kaplan–Meier method and compared by the log-rank test. OS was defined as the time from the date of blood collection to death. Multivariate survival analysis was performed utilizing the Cox proportional hazard model. Dichotomization of the level of PD-L1^+  ^CD8^+^ cells was performed by means of ROC analysis maximizing Youden’s statistics using R software. A value of 0.1505% was set as a cut-off (sensitivity 91.7%, specificity 48.3%).

### Data availability

The dataset generated during and/or analysed for the current study are available from the corresponding author on reasonable request.

## Electronic supplementary material

Supplementary Information
