# Supplementary material for: Challenging PD-L1 expressing cytotoxic T cells as a predictor for response to immunotherapy in melanoma
Source: Nat Commun. 2018 Jul 26;9:2921. doi: 10.1038/s41467-018-05047-1 (PMC6062523; doi:10.1038/s41467-018-05047-1)
Supplement: Supplementary file 1 — Supplementary Information [file 41467_2018_5047_MOESM1_ESM.pdf]

Supplementary Table 1: Patient characteristics

Number of patients, n		55
Age at diagnosis, years (median - IQR)		53 (36 - 65)
Female/male ratio (%)		51/49
Stage at diagnosis, %		
Stage I	Stage IA	10.9
	Stage IB	29.1
Stage II	Stage IIA	16.4
	Stage IIB	10.9
	Stage IIC	5.5
Stage III	Stage IIIA	9.1
	Stage IIIB	16.4
	Stage IIIC	1.8
Follow-up time since diagnosis, months (median - IQR)		61.0 (38.0 - 112.0)
Evolution during follow-up		
	disease relapse, % (n)	27.3 (15/55)
	melanoma related death, % (n)	10.9 (6/55)
Stage at inclusion, %		
Stage I	Stage IA	7.3
	Stage IB	27.3
Stage II	Stage IIA	10.9
	Stage IIB	7.3
	Stage IIC	5.5
Stage III	Stage IIIA	9.1
	Stage IIIB	21.8
	Stage IIIC	10.9
Time of inclusion, months (median - IQR)		15.0 (4.0 - 60.0)
Follow-up time since inclusion, months (median - IQR)		46.0 (22.0 - 55.0)
Evolution during follow-up after inclusion		
	disease relapse, % (n)	10.9 (6/55)
	melanoma related death, % (n)	10.9 (6/55)
